# Microcephaly models in the developing zebrafish retinal neuroepithelium point to an underlying defect in metaphase progression

**DOI:** 10.1098/rsob.130065

**Published:** 2013-10

**Authors:** Claire Novorol, Janina Burkhardt, Kirstin J. Wood, Anila Iqbal, Claudio Roque, Nicola Coutts, Alexandra D. Almeida, Jie He, Christopher J. Wilkinson, William A. Harris

**Affiliations:** 1 Department of Physiology, Development and Neuroscience, Cambridge University, Cambridge CB2 3DY, UK; 2 Centre for Biomedical Sciences, School of Biological Sciences, Royal HollowayUniversity of London, London TW20 0EX, UK; 3 PhD Programme in Experimental Biology and Biomedicine (PDBEB), Centre for Neuroscience and Cell Biology, University of Coimbra, Portugal

**Keywords:** zebrafish retina, microcephaly, STIL, ASPM, WDR62, ODF2

## Abstract

Autosomal recessive primary microcephaly (MCPH) is a congenital disorder characterized by significantly reduced brain size and mental retardation. Nine genes are currently known to be associated with the condition, all of which encode centrosomal or spindle pole proteins. MCPH is associated with a reduction in proliferation of neural progenitors during fetal development. The cellular mechanisms underlying the proliferation defect, however, are not fully understood. The zebrafish retinal neuroepithelium provides an ideal system to investigate this question. Mutant or morpholino-mediated knockdown of three known MCPH genes (*stil*, *aspm* and *wdr62*) and a fourth centrosomal gene, *odf2*, which is linked to several MCPH proteins, results in a marked reduction in head and eye size. Imaging studies reveal a dramatic rise in the fraction of proliferating cells in mitosis in all cases, and time-lapse microscopy points to a failure of progression through prometaphase. There was also increased apoptosis in all the MCPH models but this appears to be secondary to the mitotic defect as we frequently saw mitotically arrested cells disappear, and knocking down p53 apoptosis did not rescue the mitotic phenotype, either in whole retinas or clones.

## 2. Introduction

Within the central nervous system, production of the correct number of neurons from a pool of progenitor cells requires tight regulation of neural proliferation. The genes associated with autosomal recessive primary microcephaly (MCPH) are thought to be key regulators of this process. MCPH is characterized by a significant reduction in brain volume (greater than 3 standard deviations below the mean for age and sex) associated with mental retardation [1]. The cerebral cortex is disproportionately small compared with other brain structures [2]. MCPH is a rare autosomal recessive condition and is genetically heterogeneous. Since 2002, nine different causative genes have been identified: *microcephalin* [3], *aspm* [4–6], *stil* [7], *cdk5rap2/cep215* [8], *cenpj/cpap* [8–10], *cep152* [11,12], *cep63* [13], *cep135* [14] and *wdr62* [15–19].

MCPH genes are expressed at high levels in the proliferating neuroepithelium of the developing mammalian brain [3,17,20,21]. The reduced brain size in affected individuals is thought to result from a reduction in total neuron number caused by reduced proliferation of neural progenitors during fetal development [6,22]. Interestingly, all nine genes encode proteins that localize to the centrosome or spindle pole [16,17,23], highlighting the importance of centrosomes in neuronal proliferation and suggesting that there might be a common cellular mechanism underlying MCPH.

A widely supported hypothesis for the MCPH phenotype is that a premature switch from symmetric proliferative divisions to asymmetric neurogenic divisions occurs during development of the brain, leading to a reduction in the total number of neurons produced. Indeed, RNAi knockdown of *aspm* in the neuroepithelium of developing mice causes a deviation of the cleavage plane of proliferative neuroepithelial progenitors, leading to unequal inheritance of the apical membrane by daughter cells [21]. Similar findings have been reported in *microcephalin* knockout mice [24] and *cdk5rap2* mouse mutants [25]. Abnormalities in asymmetric division have also been observed in the larval brain of *Drosophila asp* mutants [26] and in *Drosophila cnn* mutants [27]. A non-mutually exclusive possibility is that MCPH mutations may lead to defective cell-cycle progression in neural progenitors, causing them to undergo fewer proliferative divisions during the crucial early stages of brain development and growth. Indeed, several recent studies have demonstrated disorganized mitotic spindles, delayed mitotic entry, mitotic arrest and reduced cell proliferation following knockdown of MCPH genes in cultured cells and animal models [28–35]. Abnormalities in centrosome inheritance have also been suggested as a possible underlying mechanism in the light of evidence that centrosome inheritance may influence neural cell fate decisions [36]. Thus, while we have gained major insights into MCPH genes over recent years, there is not yet agreement about the precise cellular mechanisms or whether there is a single underlying aetiology.

As an outpocketing of the neuroepithelium, the retina is part of the CNS. It provides many advantages for studying the neurodevelopmental roles of genes *in vivo*. For example, in the zebrafish retina, owing to its anatomical positioning and relative transparency, it is possible to make detailed *in vivo* movies of cells dividing and differentiating [37,38]. It is even possible to follow the phases of the cell cycle *in vivo* [39]. In 2007, a loss-of-function mutation in a zebrafish homologue of the human MCPH gene, *stil*, was shown to result in defective mitotic progression and increased apoptotic cell death [34]. More recently, a similar phenotype was noted in *aspm* knockdown zebrafish embryos [35]. To learn more about how these genes interfere with proliferation in the CNS, we performed functional studies of zebrafish MCPH gene homologues *stil*, *aspm* and *wdr62* in the zebrafish retina. We also studied *odf2*, the homologue of the human centrosomal gene of the same name. Although the gene encoding ODF2 is not currently linked to microcephaly, the protein is linked to several microcephaly proteins and involved in cellular processes proposed to be deficient in microcephaly. In the absence of ODF2, cell cycle progression is inhibited [40], and spindle defects are observed, similar to those caused by ASPM depletion. ODF2 interacts with Pericentrin [40], linking this protein to both CDK5RAP2 [41] and the DNA damage response in which Microcephalin is involved [42–44]. ODF2 is also a centriolar appendage protein like Ninein, whose inheritance has been claimed to be critical in asymmetric neurogenic divisions [36]. This suggested that depletion of ODF2 would also give a microcephalic phenotype when depleted from zebrafish embryos. (See electronic supplementary material, box S1 for more background on these four genes.)

We investigated the neurodevelopmental effects of morpholino-mediated knockdown of *stil*, *aspm*, *wdr62* and *odf2* in the developing zebrafish retina. We also characterized the retinal phenotype of two *stil* mutant lines, *csp*^cz65^ and *stil*^hi1262Tg^. As our findings show similar abnormalities in prometaphase progression in all of these cases, we suggest that there may be a common cellular mechanism underlying the MCPH phenotype.

## 3. Material and methods

### 3.1. Bioinformatic analysis

To identify zebrafish orthologues to human genes, NCBI protein BLAST (Basic Local Alignment Search Tool) searches were performed against the zebrafish (*Danio rerio*) proteasome. Human STIL, ASPM, WDR62 and ODF2 protein sequences were used as search queries. Additional BLAST searches were performed against the mouse (*Mus musculus*) and fruitfly (*Drosophila melanogaster*) protein databases to identify conserved domains. Multiple sequence alignment of orthologous proteins were performed using ClustalW2.

### 3.2. Animals

Zebrafish were maintained and bred at 26.5°C. Embryos were raised at 28°C and staged based on hours postfertilization (hpf) [45]. All animal work was approved by the Local Ethical Review Committee at the University of Cambridge and was conducted according to the protocols of project licence PPL 80/2198, approved by the UK Home Office.

### 3.3. Mutant and transgenic zebrafish lines

*stil*^cz65+/−^ and *stil*^hi1262Tg+/−^ zebrafish were obtained from the Zebrafish International Resource Centre (ZIRC), University of Oregon. Both mutant lines have been described previously [34]. The transgenic line *Tg*(*Fucci:GFP*) has previously been described [46] and the *Tg*(*centrin:GFP*) line was created using the pCJW266 plasmid, where the beta-actin promoter drives the expression of zebrafish centrin fused to green fluorescent protein (GFP), all flanked by ISce-1 sites [47]. *stil*^cz65+/−^ embryos were bred with *Tg*(*Fucci:GFP*), *Tg*(*H2B:GFP*) and *Tg*(*centrin:GFP*) lines to create transgenic *stil* mutant zebrafish lines.

### 3.4. Morpholino injections

Morpholino (Mo) oligonucleotides (Genetools LLC) were reconstituted as 1 mM stock solutions in water (see electronic supplementary material, table S1) and injected into the yolk sac of one-cell embryos using a Picospritzer microinjector and a micromanipulator-mounted micropipette.

We performed RT-PCR to identify whether morpholinos were acting on their target genes as predicted. A band shift was noted following injection of the anti-*aspm* morpholino (see electronic supplementary material, figure S2G) and the anti-*odf2* morpholino (see electronic supplementary material, figure S2H), reflecting disruption of the targeted genes. No band shift was noted following injection of the anti-*stil* or anti-*wdr62* morpholinos (see electronic supplementary material, figure S2F). However, the predicted action of these morpholinos was to cause exon skipping and a frame shift leading to a downstream premature STOP codon. Therefore, this lack of band shift may reflect instability of the morphant mRNA preventing successful PCR of the new product rather than inefficacy as the *stil* and *wrd62* morphants showed phenotypes that were strikingly similar to the *aspm* and *odf2* mutants and morphants.

### 3.5. Whole-mount embryo imaging

Live embryos were anaesthetized with 0.4 mg ml^−1^ MS222 (Sigma), placed in dishes containing 1.5% agarose and visualized using a dissecting stereomicroscope (Leica MZ FLIII) equipped with a QImaging micropublisher 5.0 RTV colour camera. Images were acquired using the QCapture Pro software and processed with Adobe Photoshop software.

### 3.6. Cryosections and immunohistochemistry

Whole embryos were fixed in 4% paraformaldehyde (PFA) in PBS (overnight at 4°C), rinsed in PBS, cryoprotected with 30% sucrose in PBS, embedded in Tissue-Tek OCT (Sakura) and cryosectioned at 12 µm thickness. Immunostaining of sections was performed using standard methods. Cryosections were washed in PBS (1 × 5 min) and incubated in blocking solution (1% BSA, 0.5% Triton, 10% HIGS in PBS) for 30 min at room temperature (RT). Primary antibodies, rabbit anti-phosphohistone-H3 (ser-10) (06-570, Millipore; 1 : 500) and rabbit antiactivated caspase-3 (559565, BD Biosciences; 1 : 500), were added in blocking solution and incubated overnight at 4°C. Sections were washed in PBS (6× 5 min) and incubated in secondary antibody (anti-rabbit Alexa-594 (Invitrogen, 1 : 500) or anti-mouse Alexa-488 (Invitrogen, 1 : 500)) in blocking solution (3% BSA and 0.5% Triton in PBS) for 2–3 h at RT. Sections were counterstained with 4’,6-diamidino-2-phenylindole (DAPI) in PBS and mounted with coverslips using Fluorsave mounting medium (Calbiochem).

### 3.7. Visualization and analysis of retinal sections

Cryosections were imaged with a Nikon Eclipse 80 I microscope equipped with Hamamatsu ORCA-ER colour camera and processed using Openlab software. Retinal area was measured using Openlab software. Manual cell counting in the retina, somites, spinal cord, pectoral fins and skin was performed using a standardized procedure.

### 3.8. Confocal time-lapse imaging

Control, *stil* and *odf2* morphant and *centrin-GFP stil* mutant embryos were injected with 50–100 pg of capped mRNAs (H2B : GFP or H2B : RFP) at the one-cell stage. Embryos were kept at 28°C in embryo medium supplemented with 0.003% 1-phenyl-2-thiourea (PTU) (Sigma). Imaging of live embryos was performed as previously described [34]. Live 28–30 hpf embryos were mounted on a coverslip serving as the bottom of a 30 or 50 mm Petri dish, allowing for imaging using an inverted microscope. Embryos were mounted in a 50 : 50 mixture of 1.2% low melting point agarose and embryo medium, containing MS222 (0.4 mg ml^−1^, Sigma) and 1× Steinberg solution (pH 7.4). Imaging was performed using an Olympus FV100 confocal microscope using a 60× (1.3 NA) oil immersion objective. Optical sections at 1 μm separation were taken, covering a total volume within the retina of 20–35 µm thickness. Frames were captured every 6 min for a total time of 3 h per movie.

### 3.9. Analysis of time-lapse imaging data

Confocal data was analysed using Volocity (Improvision) and ImageJ/Fiji (NIH). Cells that entered mitosis after the movie was commenced and any time up to 60 min prior to the end of the movie were identified and counted manually. Their progression through the cell cycle was followed through serial frames and the outcomes of these cells categorized. The time for cells to complete the division was recorded. Confocal z-slices were cropped to a rectangular region containing the cells of interest in XYZ. Brightness and contrast were adjusted using Photoshop (Adobe). Graphs were constructed and statistical tests were performed using Prism (GraphPad).

### 3.10. Transplantations and clonal analysis

For blastomere transplantations, *Tg*(*H2B:GFP*) donor embryos and AB host embryos were injected with control Mo, *stil* Mo, *aspm* Mo or *wdr62* Mo (+/– *p53* Mo) at the one- to two-cell stage. At 3.5–4.5 hpf, embryos were dechorionated using 0.6 mg ml^−1^ pronase (Roche) and placed in agarose moulds. A total of 5–10 blastomeres were transferred from donors into host embryos using a glass capillary connected to a 2 ml syringe. Host embryos were raised at 28°C–32°C in embryo medium supplemented with 0.003% PTU. The retinas of host embryos were screened for GFP-expressing cells at 24 hpf using an upright fluorescence microscope. The number and position of GFP-expressing cells in each retina was noted. Embryos were fixed at 48 hpf and visualized with an Olympus FV100 confocal microscope, using a 60× (1.3 NA) oil immersion objective. Confocal data were analysed using Volocity (Improvision), ImageJ/Fiji and Photoshop (Adobe).

### 3.11. RNA extraction and reverse-transcription polymerase chain reaction

RNA was extracted from whole embryos using the RNeasy Micro Kit (QIAGEN, Crawley, West Sussex, UK). RNA concentration was quantified using the spectrophotometer NanoDrop 1000 (Thermo Fisher Scientific, Wilmington, Delaware, USA). The QIAGEN OneStep RT-PCR Kit was used to perform highly sensitive and specific RT-PCR reactions. Primers (Sigma-Aldrich, Dorset, UK) used are listed in the electronic supplementary material, table S2.

Optimal RT-PCR reaction conditions were: Step 1—50°C, 30 min; Step 2—95°C, 15 min; Step 3—94°C, 30 s; Step 4—60°C, 30 s; Step 5—72°C, 2 min; nRepeat Steps 3–5 × 35; Step 6—72°C, 1 min; Step 7—4°C, end.

To exclude the possibility that RNA samples were contaminated with DNA, each reaction was also performed without the 50°C 30 min RT step. Gel electrophoresis was performed on a 1% agarose gel.

### 3.12. *In situ* hybridization

Digoxigenin-labelled RNA probes for *in situ* hybridization (ISH) were made with standard methods using sequence-verified IMAGE clones for *Danio rerio stil* (IMAGE ID: 7147918) and *aspm* (IMAGE ID: 7284669) (Geneservice, Source BioScience UK Ltd). Wild-type zebrafish embryos were fixed at 24, 48 and 72 hpf in 4% PFA in PBS (1 h at RT). Embryos were washed in PBS, placed in 30% sucrose in PBS (1 h at RT) for cryoprotection and embedded in Tissue-Tek OCT (Sakura). ISH was performed on 16 µm cryosections according to a modified version of the protocol written for *Xenopus laevis* [48]. Imaging was performed using a Zeiss Axioskop2 microscope with a Micropublisher 5.0 RTV camera and QcapturePro software. Images were processed in Photoshop (Adobe).

## 4. Results

### 4.1. Identification of zebrafish homologues to STIL, ASPM and WDR62

**Figure 1.**
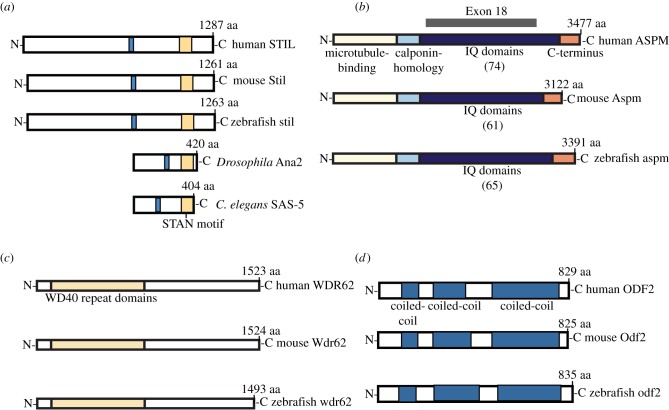
Zebrafish MCPH orthologues. Orthologues of MCPH proteins (*a*) STIL, (*b*) ASPM and (*c*) WDR62 and (*d*) centrosomal protein ODF2. Multiple species alignment demonstrates conserved domains including: the STAN motif (yellow) in STIL orthologues; the microtubule-binding domain (yellow), calponin homology domain (pale blue), IQ repeats (dark blue) and a C-terminal region of unknown function (orange) in ASPM orthologues; a WD40 repeat domain (yellow) in WDR62 orthologues; and three coiled-coil domains in ODF2 orthologues. Of note, the number of ASPM IQ domains differs between species (shown in parentheses) and exon 18 (targeted by our zf anti-*aspm* morpholino) accounts for the majority of these repeats.

To identify the zebrafish homologues to human STIL (NCBI ID: NP_001041631.1), ASPM (NCBI ID: NP_060606), WDR62 (NCBI ID: NP_001077430) and ODF2 (NCBI ID:NP_702914.1), BLAST searches were performed within the zebrafish (*Danio rerio*) protein database. Clustal analysis was performed to identify protein similarity and conserved domains. The 1263 amino acid (aa) zebrafish STIL protein was identified (NCBI ID: NP_775351.1) along with the corresponding 4817 bp *stil* gene (NCBI ID: NM_173244.1), which is located on chromosome 22 and contains 16 exons. Sequence alignment demonstrated 37% identity and 51% similarity with human STIL. Multiple sequence alignment of these proteins along with mouse, *Drosophila* and *Caenorhabditis elegans* homologues demonstrated conservation of the STAN motif across species (figure 1*a*).

The 3388 aa zebrafish ASPM protein was identified (NCBI ID: XP_003201115.1) along with the corresponding 10 523 bp *aspm* gene (NCBI ID: XM_ 003201067), located on chromosome 22 and containing 29 coding exons. Sequence alignment demonstrated 39% identity and 59% similarity with human ASPM. Multiple sequence alignment of these proteins as well as the mouse homologue demonstrated conservation of the microtubule-binding domain, calponin homology domain and multiple IQ-repeats in these proteins (figure 1*b*).

The 1519 aa zebrafish WDR62 protein was identified (NCBI ID: XP_699579.3) along with the corresponding 5204 bp zebrafish *wdr62* gene (NCBI ID: 570949), which is located on chromosome 15 and contains 31 exons. Sequence alignment demonstrated 33% identity and 42% similarity with human WDR62. Multiple sequence alignment of these proteins as well as the mouse homologue demonstrated conservation of the WD40 repeat domain across the three species (figure 1*c*).

The 831 aa zebrafish ODF2 protein (NCBI ID: XP_001332564.4) shows 52% identity and 72% similarity to human ODF2 (NCBI ID:NP_702914.1). The *odf2* gene is located on chromosome 21 and contains 19 exons. A schematic of these proteins and the mouse homologue shows the location of the coiled-coil domains in each (figure 1*d*).

### 4.2. Expression of zebrafish *stil*, *aspm*, *wdr62* and *odf2* during early development

Moderate ubiquitous expression of zebrafish *stil*, with higher expression in the head and eye regions, has previously been demonstrated by ISH in 24 hpf whole-mount embryos [34]. ISH at 24 hpf has also demonstrated strong expression of *aspm* in the zebrafish retina and CNS [35], with expression becoming largely confined to regions of high proliferation within the brain and the ciliary marginal zone (CMZ) of the retina by 48 hpf. We performed RT-PCR analysis and confirmed expression of *stil*, *aspm* and *wdr62* in wild-type zebrafish embryos at 24, 48, 72 and 96 hpf (see electronic supplementary material, figure S1A). RT-PCR also confirmed *odf2* expression in early stage zebrafish embryos (see electronic supplementary material, figure S2H). ISH was then performed for two of these genes, *aspm* and *stil*, on histological sections of wild-type embryos fixed at 24, 48 and 72 hpf (see electronic supplementary material, figure S1B). At 24 hpf, both genes are expressed throughout the retina. However, at 48 hpf the expression within the retina is more restricted, with the strongest expression noted at the CMZ. By 72 hpf, both *stil* and *aspm* are almost exclusively expressed at the CMZ (shown at higher magnification in electronic supplementary material, figure S1C). We also noted expression of both genes in the developing zebrafish brain. Again, expression was the strongest in regions containing many proliferating cells, including the periventricular regions. Expression was largely confined to these regions by 72 hpf (see electronic supplementary material, figure S1D).

### 4.3. Knockdown of zebrafish *stil*, *aspm*, *wdr62* and *odf2* causes an MCPH-like phenotype

**Figure 2.**
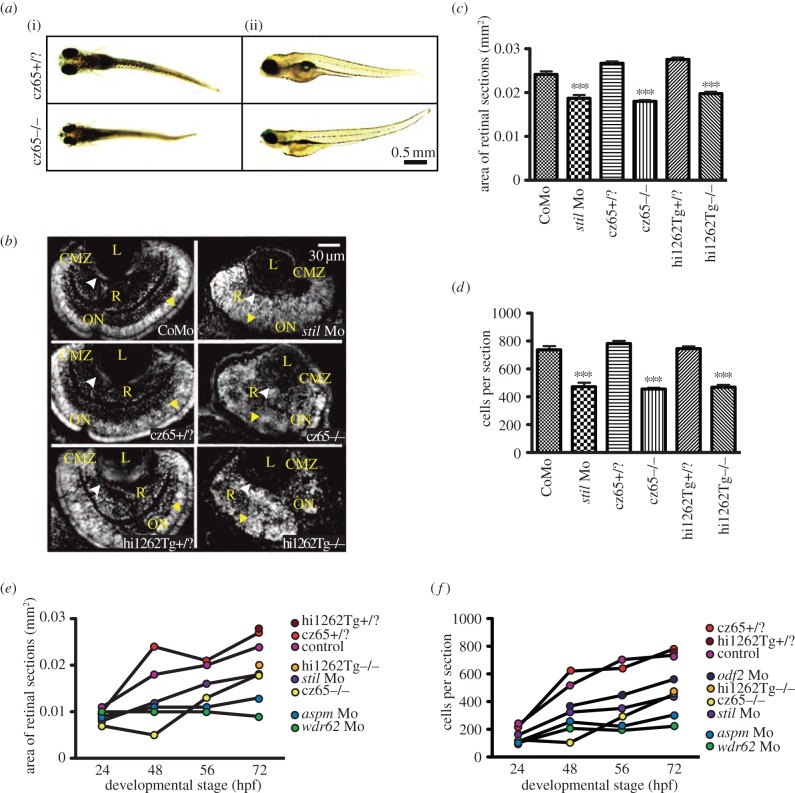
MCPH phenotypes in the zebrafish retina. Knockdown of MCPH genes causes a reduction in head size, retinal size and retinal cell number. (*a*) Reduction in head and eye size is demonstrated in whole-mount *stil*^cz65−/−^ mutant embryos on day 5 of development. Healthy *stil*^cz65+/?^ embryos are shown at the same developmental stage for comparison. While head and eye size were consistently reduced, overall body size/length of mutant embryos was variably affected. Here, one mutant (i) is smaller than the healthy embryo, whereas the other (ii) is similar in length and size to the healthy embryos. Note also the abnormally protruding lenses in the mutant embryo seen from (i), exposed owing to reduced retinal size. (*b*) DAPI-stained sections demonstrate reduced retinal area in *stil* morphants (*stil* Mo) at 72 hpf when compared with control morphants (CoMo). Similarly, the retinas of *stil*^cz65−/−^ and *stil*^hi1262Tg−/−^ mutant embryos at 72 hpf are markedly reduced in size. Labels in yellow: L, lens; R, retinal neuroepithelium; CMZ, ciliary marginal zone; ON, optic nerve; AM, apical membrane. (*c*) Retinal area is significantly reduced in *stil* morphant and mutant embryos: *stil* Mo 0.019 mm^2^ (*n* = 23) versus CoMo 0.024 mm^2^ (*n* = 34), *p* < 0.001; *stil*^cz65−/−^ 0.019 mm^2^ (*n* = 23) versus *stil*^cz65+/?^ 0.027 mm^2^ (*n* = 72) *p* < 0.001; *stil*^hi1262Tg−/−^ 0.020 mm^2^ (*n* = 52) versus *stil*^hi1262+/?^ 0.028 (*n* = 48), *p* < 0.001 (values are for mean area at 72 hpf). (*d*) Retinal cell number is reduced in *stil* morphants and mutants: *stil* Mo 471 cells (*n* = 23) versus CoMo 735 cells (*n* = 7), *p* < 0.001; *stil*^cz65−/−^ 454 cells (*n* = 114) versus *stil*^cz65+/?^ 780 cells (*n* = 72), *p* < 0.001; *stil*^hi1262Tg−/−^ 468 cells (*n* = 52) versus *stil*^hi1262+/?^ 744 cells (*n* = 48), *p* < 0.001 (values are for mean number of cells in central retinal sections at 72 hpf). (*e*) Retinal area increases as development progresses in *stil*, *aspm*, *wdr62* and *odf2* morphant embryos but remains reduced compared with control at all time-points examined (24, 48, 56 hpf at 72 hpf). (*f*) Retinal cell increases as development progresses in *stil*, *aspm*, *wdr62* and *odf2* morphant embryos but remains reduced compared with control at all time-points examined (24, 48, 56 hpf at 72 hpf), *n* = number of eyes analysed.

To investigate the phenotype associated with *stil* knockdown, we were able to take advantage of two previously characterized loss-of-function mutants, *csp*^cz65−/−^ and *stil*^hi1262Tg−/−^ (see electronic supplementary material, figure S2A). We also designed antisense morpholinos against all four genes of interest (see electronic supplementary material, figure S2B–E). Both mutants and all four morphants exhibited a consistent MCPH-like phenotype involving marked reduction in head size (figure 2*a* and not shown) and eye size (see electronic supplementary material, figure S2A–E), which became increasingly obvious as the development progressed from 24 hpf through to 72 hpf (figure 2*d*–*e*). Other abnormalities noted in some but not all mutant and morphant embryos included dorsal or ventral tail curvature, cardiac oedema and a reduction in overall size of the embryo (figure 2*a* and not shown). Examination of DAPI-stained histological sections at 24, 48, 56 and 72 hpf revealed a significant reduction in retinal size and cell number in all mutant and morphant conditions when compared with control embryos. As well as reduced retinal size, morphant and mutant embryos typically lacked the normal retinal lamination patterns apparent in wild-type embryos at 56–72 hpf suggesting possible delayed development (figure 2*a*). We also noted in severely disorganized retinas of mutant embryos that there were patchy areas of increased fluorescence suggestive of cell debris.

### 4.4. Knockdown of zebrafish MCPH genes results in metaphase delay

**Figure 3.**
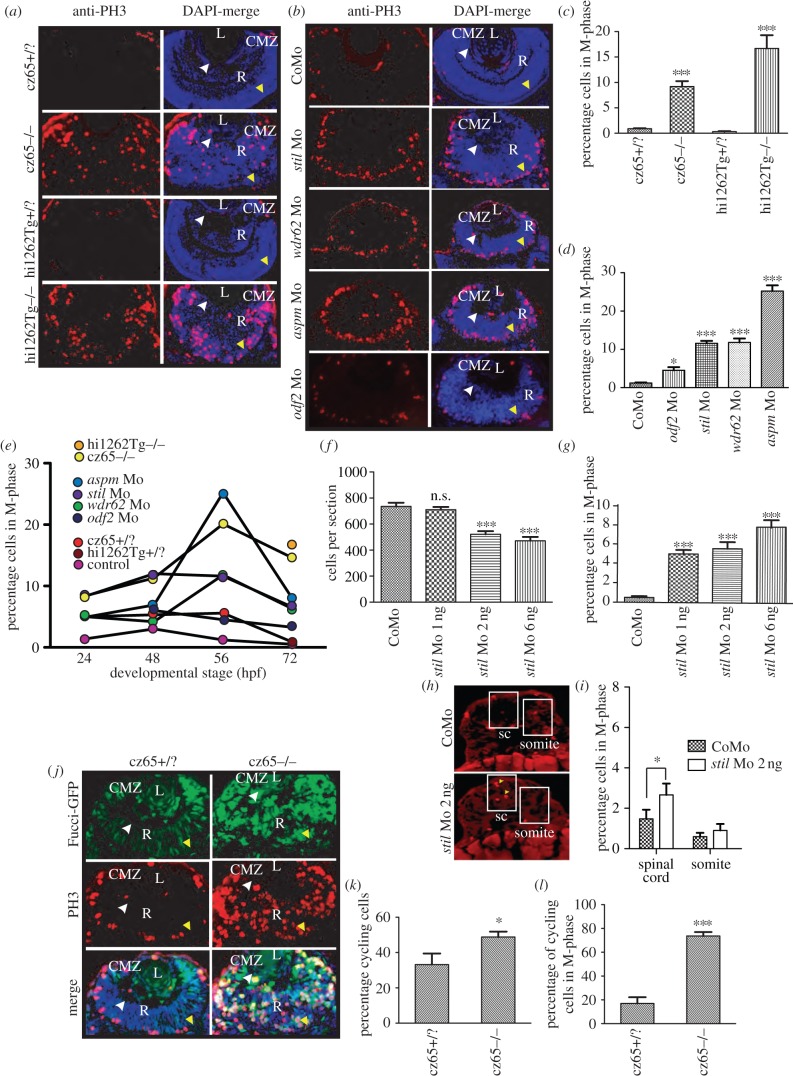
Knockdown of *stil*, *aspm*, *wdr62* or *odf2* results in an increase in mitotic cells within the developing zebrafish retina. (*a*) PH3 staining (red) reveals a dramatic increase in mitotic cells within the retina of *stil*^cz65−/−^ versus *stil*^cz65+/?^ embryos at 72 hpf and in *stil*^hi1262Tg−/−^ versus *stil*^hi1262+/?^ embryos. Mitotic cells were also abnormally localized in mutant embryos, being scattered throughout the retina rather than limited to the CMZ. DAPI-counterstain (blue) demonstrates the smaller retinas in mutant embryos, with a disorganized appearance and delayed lamination. (*b*) PH3 staining (red) demonstrates increased mitotic cells within *stil*, *wdr62*, *aspm* and *odf2* morphant retinas at 56 hpf. Note that mitotic cells are localized to the apical membrane in morphant retinas, rather than scattered throughout the retina, although they are not restricted to the CMZ as in control embryos. DAPI-counterstain (blue) also demonstrates reduced retinal size and delayed lamination. (*c*) The percentage of retinal cells in mitosis (mitotic index; MI) was significantly increased in *stil*^cz65−/−^ versus *stil*^cz65+/?^ retinas: 14.6% (*n* = 71) versus 0.9% (*n* = 56), *p* < 0.001. A similar increase was observed in *stil*^hi1262Tg−/−^ versus *stil*^hi1262+/?^ retinas: 16.7% (*n* = 32) versus 0.3% (*n* = 29), *p* < 0.001 (values are for MI at 72 hpf). (*d*) A significant increase in MI was also seen in morphant embryos (values reflect MI at 56 hpf): *odf2* Mo (4.5%; *n* = 14) versus control Mo (1.2%; *n* = 61), *p* < 0.05; *stil* Mo (11.6%; *n* = 12), *p* < 0.001; *wdr62* Mo (11.8%; *n* = 13), *p* < 0.001); and *aspm* Mo (25.2%; *n* = 25), *p* < 0.001. (*e*) An increase in the MI was observed at all examined time-points for all mutant and morphant conditions. Here, MI is plotted against developmental time-points (24, 48, 56 and 72 hpf) for each condition. Note the peak of MI around 56 hpf in most mutant and morphant conditions and the slightly earlier peak at 48 hpf in control embryos. (*f*) Reduced amounts of *stil* Mo led to a similar but less severe reduction in the number of cells per retinal section: *stil* Mo 6 ng (471 cells, *n* = 23), versus 735 *p* < 0.001; *stil* Mo 2 ng (521 cells, *n* = 17), versus 735 *p* < 0.001); *stil* Mo 1 ng (710 cells; *n* = 13) versus 735 cells in control embryos (*n* = 7), *p* > 0.05). (*g*) Reduced amounts of *stil* Mo also led to a corresponding reduction in the MI: *stil* Mo 6 ng 7.7% (*n* = 12), versus 0.5% *p* < 0.001; *stil* Mo 2 ng 5.5% (*n* = 17), versus 0.5% *p* < 0.001); *stil* Mo 1 ng 5.0% (*n* = 13) versus 0.5% in control embryos (*n* = 15), *p* < 0.001). (*h*) PH3 staining (yellow arrowheads) of spinal cord (sc) and somite tissue in control embryos and embryos injected with 2 ng anti-*stil* morpholino. (*i*) Comparison of the percentage of cells in M-phase in spinal cord and somites (*n* = 21 anti-*stil* morpholino versus *n* = 24 control morpholino embryos). (*j*) Fucci-GFP expression (green) in cycling cells, combined with anti-PH3 immunostaining (red) of fixed sections and DAPI-counterstain (blue) demonstrates an increase in the percentage of cells in *stil*^cz65−/−^ retinas that are in the cell cycle at 32 hpf and a marked increase in the percentage of those cycling cells that are in mitosis. Note also the distribution of cycling cells is abnormal, with most cycling cells (green) localized in the CMZ in control embryos but throughout the retina in mutants. Furthermore, mitotic cells (red) are seen only at the apical membrane in control retinas but throughout the retina in mutants. (*k*) The percentage of retinal cells in the cell cycle (excluding G1) is increased in *stil*^cz65−/−^ (49%; *n* = 14) versus *stil*^cz65+/?^ embryos (33%; *n* = 5), *p* < 0.01). (*l*) The percentage of these cycling cells in mitosis was also dramatically increased in *stil*^cz65−/−^ (74%; *n* = 14) versus *stil*^cz65+/?^ embryos (17%; *n* = 5, *p* < 0.001). *n* = number of eyes analysed. Labels: L, lens; R, retina; CMZ, ciliary marginal zone; white arrowhead, basal membrane; yellow arrowhead, apical membrane.

An increase in mitotic cells, as demonstrated by phosphohistone-H3 (PH3) staining, has previously been observed in *stil csp*^cz65−/−^ mutant zebrafish embryos [34]. A similar phenotype has also been noted following morpholino knockdown of *aspm* [35]. To confirm whether a similar mitotic phenotype occurs in the retina following MCPH gene knockdown, we performed PH3 immunostaining on retinal sections of fixed embryos at 24, 48, 56 and 72 hpf. In wild-type embryos at 24 hpf, most cells are dividing symmetrically to produce two more proliferating cells, and by 72 hpf most normally dividing cells in the central retina are likely to be undergoing their final division and proliferation is largely restricted to the CMZ [49]. In *csp*^cz65−/−^ and *stil*^hi1262Tg−/−^ mutant embryos, we observed a severe mitotic phenotype in the retina, with a dramatic increase in the percentage of cells in mitosis (figure 3*a*,*c*,*e*). Furthermore, mitotic cells were located throughout the retinal neuroepithelium (figure 3*a*), rather than confined to the apical membrane (at earlier time-points) or the CMZ (by 56–72 hpf) as in wild-type embryos (figure 3*a*). To determine whether a similar mitotic phenotype occurs following knockdown of other MCPH genes, PH3 immunostaining was performed on retinal sections from *stil*, *aspm* and *wdr62* morphants. Again, we noted a significant increase in mitotic cells at all time-points studied (figure 3*b*,*d*,*e*). A similar but less severe increase in mitotic cells was also noted in *odf2* morphants (figure 3*b*,*d*,*e*). Of note, the mitotic phenotype in all four morphants was generally less severe than that in *stil* mutants (figure 3*a*–*f*). In particular, in the morphant embryos, the excess mitotic cells were mostly restricted to the apical membrane (figure 3*b*), in contrast to the unusual localization of mitotic cells throughout the retina in mutants (figure 3*a*). This suggested that partial knockdown of these genes (as is likely with morpholino-mediated knockdown) might lead to a similar but less severe phenotype to that seen in cases where there is complete loss of function. To explore this hypothesis further, we focused on *stil* morphants and reduced the amount of anti-*stil* Mo used from the standard 6 ng that was used in all other experiments to 2 ng and then to 1 ng. At these reduced morpholino doses, a similar but less severe retinal phenotype was observed, confirming that the observed reduction in retinal size and cell number corresponds to the degree of *stil* knockdown (figure 3*f* and not shown). The severity of the mitotic phenotype also corresponded to the level of *stil* knockdown, with a less severe mitotic phenotype noted as the amount of *stil*-Mo injected was reduced (figure 3*g*).

Most microcephalies are associated with normal stature and organ size, except, of course, for the brain. To test for CNS specificity in our zebrafish model, we also quantified the effects of 2 ng of anti-*stil* Mo on somites, spinal cord, pectoral fins and skin. This partial knockdown was used as most microcephalies known are thought to be due to hypomorphic mutations. PH3 staining showed that percentage of spinal cord cells in mitosis was slightly increased (figure 3*h*,*i*), but the effect was not as big as that seen in the retina, which suggests that the spinal cord is not completely spared at this level of STIL reduction. However, there was no significant increase in the mitotic index of muscle cells in anti-*stil* versus control morphants (figure 3*h*,*i*). Nor was there a significant increase in the mitotic index in the pectoral fin (5.5% (*n* = 8) versus 4.1% (*n* = 10)) or epithelial cells (0.8% (*n* = 21) versus 1.9% (*n* = 23)) in *stil* versus control morphants. These results strongly suggest that partial knockdown of STIL can have effects that are fairly CNS specific.

Having observed this increase in mitotic retinal cells, we wondered whether the total number of cycling cells is also increased and whether a higher proportion of those cycling cells is in mitosis compared with wild-type. To explore this, we made use of a transgenic *Fucci:GFP* line, in which GFP is expressed by proliferating cells in S-phase, G2-phase or M-phase [16]. We crossed the *csp*^cz65^ *stil* mutant line with the *Tg*(*Fucci:GFP*) line and performed PH3 immunostaining on retinal sections of embryos fixed at 32 hpf. This allowed us to determine the approximate number of cycling cells as well as the number of cells in mitosis in mutant/morphant compared with healthy retinas. We observed a significant increase in the percentage of retinal cells in the cell cycle at 32 hpf in mutant embryos (figure 3*j*,*k*), from 33 to 49%. Furthermore, the percentage of those cycling cells specifically in mitosis was dramatically increased (figure 3*l*), from 17 to 74%.

### 4.5. Time-lapse analysis of the MCPH phenotype suggests prometaphase delay occurs in retinal progenitor cells, with associated problems in centrosomes

**Figure 4.**
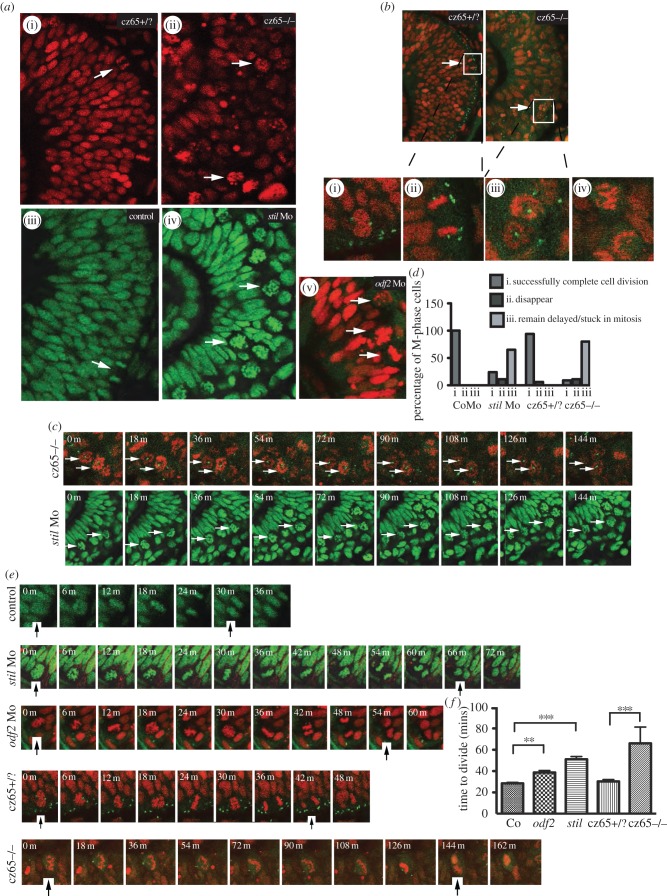
MCPH gene depletion causes a block or delay at prometaphase. Mitotic retinal cells in *stil* mutant embryos appear to be delayed in prometaphase. A similar but less severe phenotype occurs in *stil* morphants and *odf2* morphants. (*a*) Views of the retina at approximately 30 hpf demonstrate (i) the normal appearance of retinal progenitor cells in *stil*^cz65+/?^ embryos (H2B-RFP marks nuclei red) and (iii) control embryos (H2B-GFP marks nuclei green). White arrows mark dividing cells (anaphase) at the apical membrane. (ii) By contrast, in *stil*^cz65−/−^ embryos the retina appears disorganized with markedly more cells in mitosis. The appearance of these cells (white arrows) suggests they are in prometaphase. A similar but less severe phenotype was observed in (iv) *stil* morphants and (v) *odf2* morphants. In both morphant conditions, numerous ‘prometaphase’-like cells were observed in the retina (white arrows), although in contrast to mutants these cells were localized near to the apical membrane. (*b*) Centrosomal abnormalities are present in *stil*^cz65−/−^ embryo retinas, including reduced centrosome expression and loss of apical centrosome positioning. Normal apical centrosomes are seen in *stil*^cz65+/?^ embryos (green; centrin-GFP). (i) As cells round up and enter mitosis in *stil*^cz65+/?^ embryos two centrosomes can be seen. (ii) A dividing cell is shown in a *stil*^cz65+/?^ embryo, with a single centrosome at each pole of the newly forming daughter cells. By contrast, in *stil*^cz65−/−^ embryos mitotic cells lack one or both centrosomes. (iii) Many prometaphase-like cells appear to be associated with only a single centrosome (white arrows, and at high magnification in (v) or (iv) no centrosome. (*c*) Many mitotic cells in *stil*^cz65−/−^ mutants and *stil* morphants remain arrested in mitosis throughout live 2–3 h movies. Here frames demonstrate cells arrested in mitosis (white arrows) in *stil*^cz65−/−^ embryos (nuclei in red; marked by H2B-RFP; centrosomes in green; marked by centrin-GFP) over a period of at least 144 min. Over the same period, *stil* morphant cells (green; marked by H2B-GFP) are also seen arrested in mitosis (white arrows). (*d*) Throughout movies a marked reduction in the percentage of cells successfully completing division was noted in *stil* mutants and morphants, with many cells remaining delayed or stuck in mitosis. In control embryos, 100% of cells entering mitosis during the first 2 h of a 3-h movie successfully completed cell division before the end of the 3-h movie (*n* = 29). A similar outcome was observed in *stil*^cz65+/?^ control embryos; 94% of cells successfully completed cell division with 6% of cells disappearing from view (*n* = 17). In *stil* morphants, only 24% of cells successfully completed division, with 11% disappearing from view and 65% remaining arrested or delayed in M-phase for 60 min or longer (*n* = 37). In *stil*^cz65−/−^ mutants, the phenotype was even more severe, with 11% of mitotic cells disappearing, 80% remaining stuck or delayed in M-phase and only 9% successfully completing mitotic division (*n* = 54). Three separate 180-min movies were analysed for each condition. *n* = total number of mitotic cells analysed for each condition. (*e*) Successful mitotic divisions were slower in morphants and mutants versus control. Typical divisions are shown for control, *stil* morphant, *odf2* morphant, *stil*^cz65+/?^ and *stil*^cz65−/−^ embryos. Black vertical arrows indicate the beginning of M-phase (0 min), when the dividing cell rounds up at prophase, and the end of M-phase, when two daughter cells have been formed and chromatin decondensation has commenced. In these examples, mitosis took approximately 30 min (control), 66 min (*stil* Mo), 54 min (*odf2* Mo), 42 min (*stil*^cz65+/?^) and a minimum of 144 min (*stil*^cz65−/−^) (note that for the *stil*^cz65−/−^ mutant the dividing cell had already entered M-phase before the movie commenced so the true length of time to complete division was longer than this minimum estimate). (*f*) The time for morphant and mutant retinal cells to successfully complete mitotic division was increased. Three separate movies were analysed for each condition. The mean time to complete mitotic cell division was increased in *stil* morphants (*n* = 23) versus control (*n* = 29) (51 versus 29 min; *p* < 0.001) and *odf2* morphants (*n* = 55) (38.7 versus 29 min; *p* < 0.01). The mean time to complete mitotic cell division was also markedly increased in *stil*^cz65−/−^ embryos (*n* = 5) versus *stil*^cz65+/?^ (*n* = 15) (at least 66 versus 30 min; *p* < 0.001). Overall, mitotic cell division within the retina was most severely prolonged in *stil*^cz65−/−^ mutants and moderately prolonged in both *stil* morphants and *odf2* morphants. *n* = number of successful mitotic divisions analysed.

To investigate the mitotic phenotype in more detail, *in vivo* time-lapse imaging of the developing retina was performed in *stil csp*^cz65−/−^ mutants, *stil* morphants and *odf2* morphants at approximately 30 hpf. Embryos with nuclei fluorescently marked were examined at 6-min intervals during 3-h movies. In *stil* mutants, the retina was strikingly disorganized, with large numbers of ‘rounded-up’ mitotic cells scattered throughout the retina (figure 4*a*(ii)). These cells appeared to contain scattered condensed chromosomes that were not forming a metaphase plate or entering anaphase. This was in contrast to the organized appearance of the unaffected *csp*^cz65+/?^ retina (figure 4*a*(i)), in which mitotic cells (white arrows) were observed transiently and only at the apical membrane, often in metaphase or anaphase stages.

The appearance of these abnormal cells in the mutant retina suggests that they were in prometaphase. The majority of these cells (80%) did not exit this phase at any time during the entire 3 h movie (figure 4*d*) but instead remained arrested at this early stage of mitosis (figure 4*c*,*d*). A further 11% disappeared from view during the movie and only 9% successfully completed division. By contrast, in control *csp*^cz65+/?^ embryo retinas, 94% of cells that entered M-phase during the first 2 h of a 3-h time-lapse movie had successfully completed cell division before the end of that movie, with just 6% disappearing from view and no cells remaining stuck in mitosis (figure 4*d*), and in control-morpholino-injected embryos 100% of cells successfully divided (figure 4*d*). Time-lapse imaging of *stil* morphant embryo retinas demonstrated a similar but less severe phenotype to *stil* mutants (figure 4*a*(iv)) with only 24% of cells successfully completing cell division within the same time-frame (figure 4*d*). Eleven per cent of cells disappeared from view during the movie and 65% remained arrested in early mitosis. As in the mutant, the majority of these morphant cells were already arrested at what appeared to be prometaphase at the beginning of the movie and remained in this state for the full 3 h. We went on to perform similar time-lapse analysis of *odf2* morphants. In *odf2* morphants, complete mitotic arrest was not observed during any of the movies. However, many divisions were delayed at prometaphase, with the longest observed division taking 80 min, in contrast to a maximum of 36 min in control embryos (figure 4*a*(v),*d*).

While the majority of mitotic cells in both *csp*^cz65−/−^ mutants and *stil* morphants remained arrested in mitosis throughout these movies, a small number of cells did successfully complete mitotic division (figure 4*e*). All successfully completed cell divisions were analysed for each condition and the mean average length of mitosis, or time to complete division, was calculated. The average length of successful cell divisions was greater in *stil* morphants, and greater still in *csp*^cz65−/−^ mutants, than in unaffected wild-type and *csp*^cz65+/?^ embryos (figure 4*f*). Figure 4*e* shows examples of mitotic cell divisions that took 30 min (control), 66 min (*stil* Mo), 54 min (*odf2* Mo) 42 min (*csp*^cz65+/?^) and at least 144 min (*csp*^cz65−/−^). (Note that in the latter example, the cell was already in M-phase at the start of the movie so the division took a minimum of 144 min but possibly much longer.) The mean length of time taken to complete mitotic cell division was 28.5 min in control embryos, 30.4 min in *csp*^cz65+/?^ embryos, 38.7 min in *odf2* morphants, 51.1 min in *stil* morphants and over 66 min in *csp*^cz65−/−^ mutants (figure 4*f*). Therefore, not only do fewer retinal cells appear to complete mitotic cell division in MCPH morphant and mutant embryos, with a large number of cells remaining arrested at prometaphase in the more severely affected embryos, but those cells which are seen to successfully divide take longer to do so. Stages beyond metaphase (anaphase, telophase and cytokinesis) all seemed to occur at a normal or near-normal speed in the small numbers of cells in *csp*^cz65−/−^ mutants and *stil* morphants that we observed progressing through metaphase following a delay. Together, these findings suggest that overall reduction in neuroepithelial cell number is because fewer progenitor cells successfully complete mitotic cell division in the retina of these developing embryos and that the main block occurs at prometaphase.

Previous examination of cells within the tail of *csp*^cz65−/−^ embryos showed that they frequently lacked one or both centrosomes [34]. To explore this finding in the retina, we crossed the *csp*^cz65^ line with a *Tg*(*Centrin;GFP*) transgenic line in which centrosomes fluoresce green. We then injected these transgenic embryos with H2B-RFP RNA to mark cell nuclei red before performing time-lapse imaging at approximately 30 hpf. In *csp*^cz65+/?^ embryos, centrosomes were located exclusively at the apical surface of the retina (figure 4*b*) at the apical footplates of non-dividing cells (as confirmed in movies of embryos expressing GAP-GFP (not shown)). In dividing cells, centrosomes were localized to both spindle poles (seen here in high magnification in figure 4*b*(i),(ii). By contrast, loss of the normal localization of centrosomes to the apical surface was observed in the *csp*^cz65−/−^retina (figure 4*b*). Instead, mitotic cells were frequently associated with single centrosomes, located at the centre of the nucleic material (figure 4*b*(iii)), or with no centrosomal material (figure 4*b*(iv)).

### 4.6. The proliferative potential of wild-type and microcephalic retinal progenitors

**Figure 5.**
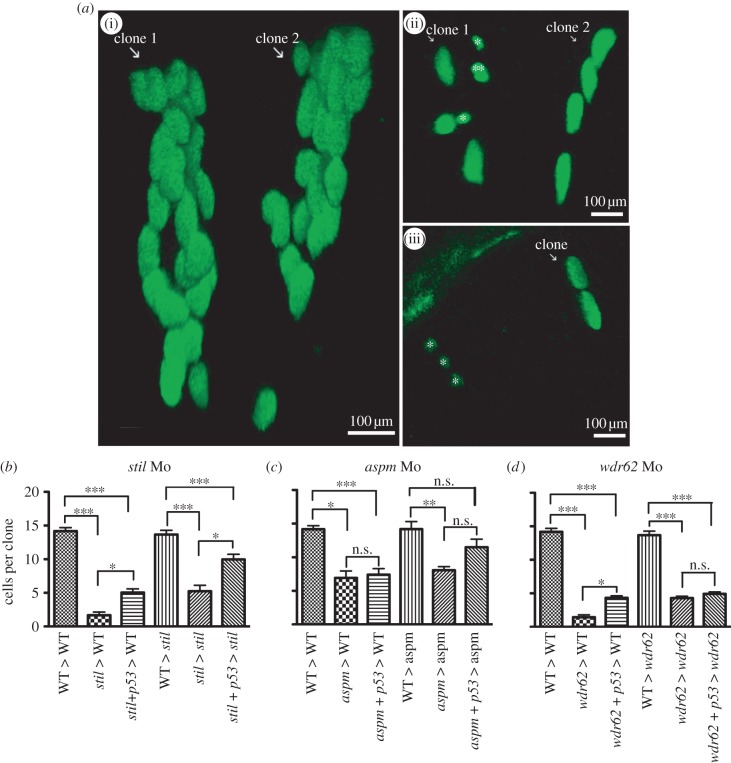
Morpholino knockdown of *stil*, *aspm* or *wdr62* led to reduced clonal proliferation of retinal progenitors *in vivo*. Blocking apoptosis only partially rescued clonal potential. Cells from H2B-GFP-expressing wild-type (WT) or morphant donor embryos were transplanted into WT or morphant host embryos at approximately 3.5 hpf. Host embryo retinas were screened for GFP-expressing one to two cell clones at 24 hpf and those clones were analysed again at 48 hpf. Graphs (*b*–*d*) show the mean cells per clone at 48 hpf (derived from a single cell at 24 hpf). The average size of retinal clones derived from WT cells in WT hosts was 14.2 cells (*n* = 73). (*a*)(i) Two typical WT clones in a WT host retina at 48 hpf, each derived from a two-cell clone identified at 24 hpf. No significant difference in clone size was seen when cells from control embryos were transplanted into WT environments (not shown): CoMo: 13.9 cells (*n* = 7) versus WT: 14.2 cells (*n* = 73) (*p* > 0.05). (*b*) *stil* morphant cells had a markedly reduced clonal capacity in WT hosts: *stil* Mo 1.7 cells (*n* = 8) versus 14.2 cells for WT (*n* = 73) (*p* < 0.001). Partial rescue of clone size was achieved with injection of anti-*p53* Mo to block apoptotic cell death: *stil* + *p53* Mo donor cells in WT hosts: 5.1 cells (*n* = 25) versus 1.7 cells without *p53* Mo (*n* = 8) (*p* > 0.05). However, clones remained significantly smaller than WT: 5.1 cells (*n* = 25) versus 14.2 cells (*n* = 73) (*p* < 0.001). A similar result was seen when WT or *stil* morphant cells were transplanted into *stil* morphant hosts. Within the morphant environment, *stil* morphant cells produced smaller retinal clones compared with WT cells: 5.2 cells (*n* = 9) versus 13.7 cells (*n* = 27) (*p* < 0.001). (*a*)(ii) A typical example of morphant cell clones within a morphant host environment at 48 hpf. GFP-expressing cells from a *stil* morphant donor were transplanted into a *stil* morphant host. Clone 1 contains seven cells with one cell (marked double asterisks (**)) presumed to be undergoing mitosis at the time of imaging. In addition, two small cells that appear to be shrinking (marked single asterisk (*)) were presumed to be undergoing apoptotic cell death. Clone 2 contains four cells. Partial rescue of clone size could be achieved by injection of anti-*p53* Mo to block apoptotic cell death: *stil* + *p53* Mo donor cells in *stil* hosts: 10.0 cells (*n* = 31) versus 5.2 cells (*n* = 9) (*p* < 0.05). However, clones remained significantly smaller than WT clones: 10.0 cells (*n* = 31) versus 13.7 cells (*n* = 27) (*p* < 0.001). (*c*) a*spm* morphant cells also produced smaller clones than WT cells; 6.9 cells (*n* = 3) versus 14.2 cells (*n* = 73) (*p* < 0.05). Partial rescue of clone size could be achieved by injection of anti-*p53* Mo: *aspm* + *p53* Mo donor cells in WT hosts: 7.4 cells (*n* = 20) versus 6.9 cells without *p53* Mo (*n* = 3) (*p* > 0.05). However, clones remained significantly smaller than WT: 7.4 cells (*n* = 20) versus 14.2 cells (*n* = 73) (*p* < 0.01). Within the morphant environment, *aspm* morphant cells produced smaller retinal clones than WT cells: 8.1 cells (*n* = 15) versus 14.2 cells (*n* = 12) (*p* < 0.01). Partial rescue of clone size could be achieved with injection of anti-*p53* Mo: *aspm* + *p53* Mo donor cells in *aspm* hosts: 11.5 cells (*n* = 14) versus 8.1 cells (*n* = 15); *p* < 0.05. However, clone size still remained smaller than WT clones: 11.5 cells (*n* = 14) versus 14.2 cells (*n* = 12) (*p* > 0.05). (*d*) *wdr62* morphant cells also produced smaller clones than WT; 1.5 cells (*n* = 12) versus 14.2 cells (*n* = 73) (*p* < 0.001). (*a*)(iii) A typical example of morphant cell clones within a WT host environment at 48 hpf. GFP-expressing *wdr62* morphant cells were transplanted into WT host embryos. Two clones are seen, derived from two single cells identified at 24 hpf. One clone (white arrow) contains two cells. The second clone consists of three small cells (marked single asterisk (*)), all presumed to be undergoing apoptotic cell death. Partial rescue of clone size was achieved by injection of anti-*p53* Mo: *wdr62* + *p53* Mo donor cells in WT hosts: 4.3 cells (*n* = 40) versus 1.4 cells without *p53* Mo (*n* = 12) (*p* < 0.05). However, clones remained significantly smaller than WT clones: 4.3 cells (*n* = 40) versus 14.2 cells (*n* = 73) (*p* < 0.001). Within the morphant environment, *wdr62* morphant cells produced smaller clones than WT cells: 4.3 cells (*n* = 26) versus 13.7 cells (*n* = 18) (*p* < 0.001). No significant rescue was achieved by injection of anti-p53 Mo to block apoptotic cell death: *wdr62* + *p53* Mo donor cells in *wdr62* hosts: 4.9 cells (*n* = 28) versus 4.3 cells (*n* = 26) (*p* > 0.05). Clone size remained significantly smaller than WT clones: 4.9 cells (*n* = 28) versus 13.7 cells (*n* = 18) (*p* < 0.001). *n* = number of surviving clones examined at 48 hpf.

To look at the proliferative potential of retinal progenitors affected by MCPH gene knockdown in more detail, we performed *in vivo* clonal analysis. Cells with GFP-marked nuclei from wild-type or morphant embryo donors were transplanted into wild-type or morphant host embryos early in development (approx. 3.5 hpf). Retinas of host embryos were then screened for GFP-expressing cells by fluorescence microscopy at 24 hpf. At this time-point, we identified clones of one or two cells to be tracked. We then found these clones again *in vivo* at 48 hpf and counted the cells. The average size of a wild-type retinal clone in a wild-type host at 48 hpf (figure 5*a*(i)) was 14.2 cells, and clones derived from control-morpholino-injected embryos gave an average clone size of 13.9 cells, demonstrating that the injection procedure had no significant effect on proliferation. When morphant cells were then transplanted into morphant hosts, clone size was considerably reduced. *stil* morphant cells (in s*til* morphant environments) (figure 5*a*(ii)) produced clones of mean size 5.2 cells, *aspm* morphant cells (in *aspm* morphant host environments) produced clones of mean 8.1 cells (figure 5*c*) and *wdr62* morphant cells (in *wdr62* morphant host environments) produced clones of mean 4.3 cells (figure 5*d*). Thus, individual morphant progenitors show a dramatic decrease in proliferation.

Recent work has suggested that zebrafish retinal progenitors lose their proliferative potential as they divide [38]. It is not known, however, whether this loss in proliferative potential is intrinsic or due to feedback from recently differentiated cells in the local environment as suggested by Cerveny *et al*. [50]. To test whether the environment was contributing to the decrease in average clone size in microcephalic retinal progenitors, we transplanted morphant cells into a wild-type environment. Surprisingly, the average clone size for *stil* morphant cells in the wild-type environment was just 1.7 cells (figure 5*b*), for *aspm* morphants (figure 5*c*) it was 6.9 cells and for *wdr62* morphants it was 1.5 cells (figure 5*a*(ii),*d*). These results suggested not only that morphant cells had intrinsically decreased proliferative potential, but also that they do even worse in a wild-type environment. It is possible that wild-type cells may actually compete with morphant cells in such an environment, perhaps by killing them, as is seen in similar competitive scenarios in *Drosophila* [51]. To give the morphant cells a better chance of survival in the wild-type environment, we injected these donors with a morpholino to the proapoptosis factor, p53. Indeed, knocking down p53 partially rescued the reduced clonal capacity of morphant cells in the wild-type host retina, such that the average clone size matched that of morphant cells transplanted into morphant host retinas (figure 5*b*–*d*). This suggests that morphant cells are indeed at a competitive disadvantage in the wild-type environment.

A negative feedback signal from differentiated cells that limits the proliferative potential of retinal progenitors [50] might mean that wild-type cells would proliferate more in a microcephalic environment. Surprisingly, perhaps, this does not appear to be the case. When wild-type cells were transplanted into morphant hosts, no significant difference in the average retinal clone size was observed compared with wild-type clones in wild-type hosts (figure 5*b*–*d*). This suggests that there is an intrinsic proliferative capacity of wild-type cells that is not increased by transplantation into an environment with fewer cells and that the transplantation of scattered, genetically corrected cells into a microcephalic brain is unlikely to provide much of a rescue.

### 4.7. Knockdown of zebrafish MCPH genes causes increased apoptotic cell death within the developing retina

**Figure 6.**
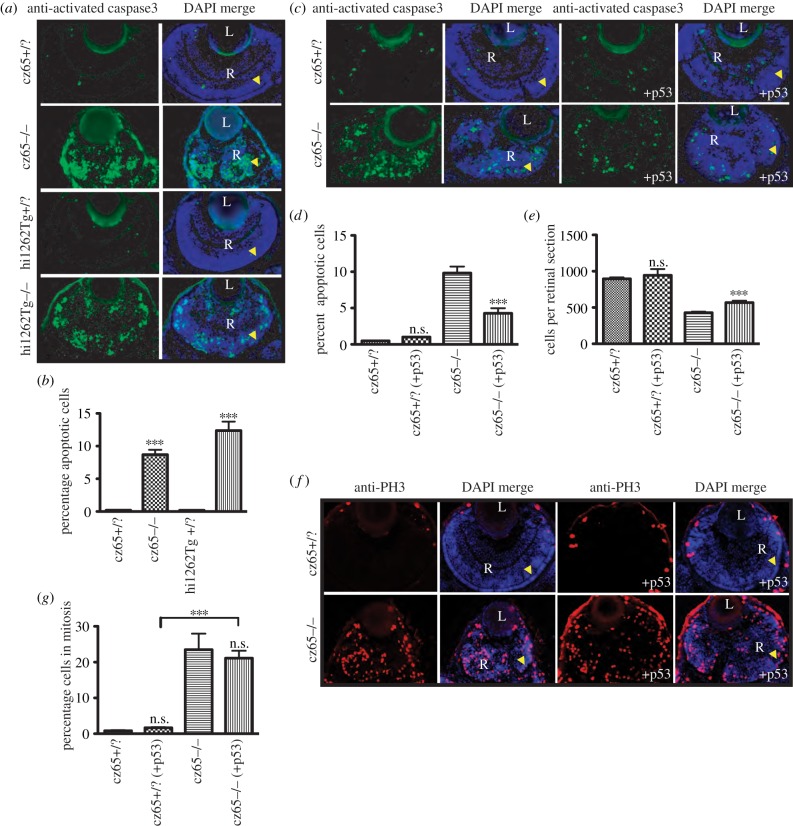
Apoptosis is associated with the MCPH phenotype. (*a*,*b*) High levels of apoptotic cell death occur within the retina of developing *stil* mutant embryos. (*a*) Little or no apoptotic cell death, as marked by antiactivated caspase-3 antibody (green) was seen in *stil*^cz65+/?^ retinas at 72 hpf (also shown with DAPI-counterstain in blue). By contrast, high levels of apoptotic cell death were seen throughout the retina of *stil*^cz65−/−^ embryos. A similar pattern was seen in *stil*^hi1262Tg−/−^ embryos, with little apoptotic death in *stil*^hi1262+/?^ retinas, but high levels of apoptosis in the *stil*^hi1262Tg−/−^ mutant. (*b*). In *stil*^hi1262Tg−/−^ mutants at 72 hpf, 8.7% of retinal cells were observed to be undergoing apoptosis (*n* = 52) versus 0.2% in *stil*^cz65+/?^ (*n* = 24) (*p* < 0.001). In *stil*^hi1262Tg−/−^ embryos, 12.4% of retinal cells were apoptotic (*n* = 16) versus 0.2% in *stil*^hi1262+/?^ (*n* = 22) (*p* < 0.001). (*c*–*g*) Blocking apoptosis in *stil* mutant embryos partially rescued the retinal phenotype but did not rescue the mitotic phenotype. (*c*–*d*) anti-*p53* Mo injection led to a reduction in apoptosis (green; antiactivated caspase-3) in *stil*^cz65−/−^ mutants at 72 hpf, from 9.8% of cells (*n* = 39) to 4.3% (*n* = 15), *p* < 0.001. By comparison, 0.47% of retinal cells underwent apoptosis in *stil*^cz65+/?^ embryos (*n* = 38) with no significant difference with anti-*p53* Mo (1.0%; *n* = 2), *p* > 0.05. This anti-*p53* Mo-related reduction in apoptosis led to partial rescue of retinal size (*c*,*e*), with an increase in mean cells per retinal section to 568 (*n* = 15) from 429 (*n* = 39), *p* < 0.01. A smaller, non-significant increase in retinal cells was seen in *stil*^cz65+/?^ embryos; 944 (*n* = 2) versus 895 (*n* = 38), *p* > 0.05). (*f*) While anti-*p53* Mo reduces apoptosis and led to an increase in retinal size and cell number, there was no significant effect on the mitotic phenotype, as demonstrated by anti-PH3 immunostaining (red). (*g*) anti-p53 Mo injection had no significant effect on MI (*stil*^cz65−/−^ with p53 Mo; 21.2% (*n* = 12) versus 23.5% (*n* = 7) for *stil*^cz65−/−^ without p53 Mo, *p* > 0.5. As a control, anti-*p53* Mo was also injected into *stil*^cz65+/?^ embryos with no significant effect on percentage of mitotic cells (*stil*^cz65+/?^ with *p53* Mo 1.7% (*n* = 13) versus 0.8% (*n* = 3) in *stil*^cz65+/?^ embryos without *p53* Mo; *p* < 0.05. *n* = number of eyes analysed.

Increased levels of apoptosis have previously been observed in *csp*^cz65−/−^ embryos by whole-mount TUNEL staining [34]. In addition, we observed significant levels of hyperfluorescent cellular debris in DAPI-stained sections of *csp*^cz65−/−^ mutants (figure 2*a*) as well as *in vivo* time-lapse movies (not shown). This led us to investigate whether apoptosis was a consistent feature of the zebrafish retinal MCPH phenotype by performing immunostaining on fixed retinal sections using anti-activated caspase-3. This confirmed high rates of apoptosis in the retina of both the *csp*^cz65−/−^ mutant and the *stil*^hi1262−/−^ mutant at 72 hpf (figure 6*a*). Whereas levels of apoptosis were very low in unaffected embryos (0.2%), 8.7% of cells stained positive for activated caspase-3 in the retinas of *csp*^cz65−/−^ mutant embryos and 12.5% in the retina of *stil*^hi1262−/−^ mutants (figure 6*b*). We were interested in elucidating to what degree this apoptotic cell death might be contributing to the observed reduction in retinal cell number. To explore this, we made use of the anti-p53 Mo. While p53 expression cannot be considered synonymous with cell death, we found that blocking *p53* activity in *csp*^cz65−/−^ mutants led to a 56% reduction in apoptotic death, from 9.8% of cells to 4.3% of cells (figure 6*c*,*d*) and to a 32% increase in mean retinal cell number (figure 6*e*). This suggests that apoptotic cell death directly leads to some of the reduction in retinal cell number observed in *stil* mutant embryos. This is consistent with our data from p53-Mo-injected morphant MCPH cells that were transplanted into morphant retinas. In these cases, the average clone size also increased by rather similar amounts (figure 5*b*–*d*). These results indicate that both an increase in apoptosis and a reduction in proliferative potential combine to cause the observed reduction in retinal clone size.

Apoptosis may well be expected if cells fail to exit metaphase or have increases in aneuploidy following imperfect spindle formation [52]. Although we did not look for aneuploidy in our experiments, we found that blocking *p53* expression had no significant effect on the mitotic phenotype, as demonstrated by anti-PH3 immunostaining of mitotic cells (figure 6*f*,*g*), suggesting that the mitotic phenotype may be physiologically upstream of the increased apoptosis. The increase in the percentage of disappearing mitotically arrested cells in our time-lapse movies also supports this chain of events.

## 5. Discussion

We have demonstrated that the MCPH gene homologues *stil*, *aspm* and *wdr62* are essential for normal progenitor proliferation in the developing zebrafish retinal neuroepithelium. MCPH gene knockdown embryos have smaller heads than wild-type embryos and smaller retinas containing fewer cells. A similar but less severe phenotype was noted following the knockdown of *odf2*, a gene encoding a centrosomal protein that is a molecular partner to Pericentrin [40], and therefore linked to MCPH genes *CDK5RAP2/CEP215* [41] and *microcephalin* [42].

The decrease in retinal cell number noted in each of these knockdowns coincided, paradoxically, with an increased mitotic index. This suggests a delay or block in mitotic progression, as has previously been noted in stil *csp*^cz65−/−^ mutants [34]. We noted that the transgenic insertion mutant *stil*^hi1262Tg−/−^ [53,54] had previously been observed to show a less severe mitotic phenotype than the *csp*^cz65−/−^ mutant, although in our study both *stil* mutants examined showed a similarly dramatic mitotic phenotype.

Through live time-lapse imaging of *csp*^cz65−/−^ *stil* mutants, *stil* morphants and *odf2* morphants during early development we confirmed that mitotic divisions are slower in the developing retinal neuroepithelium in zebrafish and that this retardation occurs during prometaphase. We did not perform time-lapse imaging for all morphant conditions, yet the appearance of excess mitotic cells on histological sections in *aspm* and *wdr62* morphants, closely resembling the phenotype noted in *stil* mutants and both *stil* and *odf2* morphants, suggests that a similar delay in early mitosis is likely to occur following knockdown of each of these genes.

We have also shown that knockdown of these MCPH genes is associated with an increase in apoptosis. Blocking p53 expression partially rescued retinal cell number, as demonstrated on fixed histological sections, although it did not affect the mitotic abnormalities. Furthermore, *stil*, *aspm* and *wdr62* morphant cells all produced smaller clones *in vivo* than wild-type cells, through a combination of reduced proliferative capacity and increased apoptotic cell death. These data indicate that both delayed mitotic progression and increased apoptotic cell death contribute to the MCPH-like phenotype observed following knockdown of these genes in zebrafish.

In this study, we have not mimicked the specific mutations that cause MCPH. As such, caution is required in extrapolating these findings to a discussion of the mechanisms that may underlie the human MCPH phenotype. MCPH is believed to be caused by hypomorphic rather than full loss-of-function mutations. Indeed, when we created a partial knockdown of STIL, we were able to mimic another feature of microcephaly, which is a preferential effect in the CNS compared with other tissues. Although there is no reason to suspect that the level of knockdown caused by injection of morpholino into the egg leads to a more pronounced reduction of the protein in the CNS, we cannot at present rule this out in our experiments. Importantly, the knockdown phenotype observed is consistent for all four genes investigated, with the most severe phenotype noted in loss-of-function *stil* mutants, and a similar but less severe phenotype in *stil*, *aspm*, *wdr62* and *odf2* morphants. These data may therefore provide clues to the possible mechanisms underlying human primary microcephaly.

The abnormal number of centrosomes and centrosome localization observed in *stil* mutant embryos might reflect defects in centrosome maturation or duplication. Indeed, this finding would be consistent with the known role of *stil* in centrosome duplication [55–57]. In mammals, it has been hypothesized that the asymmetric inheritance of centrosomes by progenitor and neuronal daughter cells may affect cell fate [36]. If this is the case, then disruption of normal centrosome expression and localization in these embryos might lead to defects in centrosome inheritance and cell fate decisions, affecting retinal neuroepithelial cell proliferation and cell-cycle exit. It was interesting to note the non-apical location of mitotic cells in *stil* mutants and to a lesser degree in the *odf2* morphant. The 2009 study by Wang [36] also observed early migration of neural progenitor cells away from the ventricular surface of the mouse neuroepithelium following loss of the centrosomal appendage protein, Ninein. Therefore, these genes may play a crucial role in anchoring cells to the neuroepithelial membrane during proliferation and controlling normal cell-cycle exit.

A particularly interesting and unresolved question is that of why it is only the brain that is affected in MCPH, despite the evidence that the MCPH genes are expressed in fetal and adult tissues throughout the body. Consistent with the human MCPH phenotype, in our mutant and morphant embryos, the head and eyes were consistently reduced in size, whereas body size was typically normal or reduced to a lesser extent. Indeed, partial gene knockdown of *stil* has a milder phenotype in the spinal cord than in the retina and much less severe phenotype in non-neuronal tissues. This echoes the phenotypes of human microcephalies, most of which primarily affect the brain. One might wonder how it is that proteins involved in a process as basic to development as mitosis could have a tissue-specific phenotype. We suggest that metaphase in progenitor cells of the human brain, and the zebrafish retina, may be particularly sensitive to defects in these genes, simply because these organs are producing more cells than most other tissues in the respective animals and the high proliferation rates cannot cope with low levels of these gene products.

Further insights in this area might be derived through gaining an understanding of why homozygous mutations within *CPAP* and *CEP152* can cause both MCPH [8], in which microcephaly is an isolated anatomical feature, and in other cases, Seckel syndrome [12,58,59], in which microcephaly occurs as part of a more extensive phenotype involving characteristic facial features and severe short stature. Detailed *in vivo* functional studies of these and other centrosomal genes in animal models should, in time, provide insights not only into the pathogenesis of these devastating conditions and the particular mechanisms that are disturbed in different cases, but also into the pathways that control normal neural proliferation during early embryonic development. Our study demonstrates that the zebrafish retinal neuroepithelium provides a valuable model system in which functional studies of MCPH and other centrosomal genes can be performed, providing insights into their normal developmental roles as well as the pathological effects of their disturbance.

## Supplementary Material

Box 1

## Supplementary Material

Supplementary Figure S1

## Supplementary Material

Supplementary Figure S2

## Supplementary Material

Supplementary figure legends

## Supplementary Material

Supplementary Table 1

## Supplementary Material

Supplementary Table 2
