# Supplementary material for: Microcephaly models in the developing zebrafish retinal neuroepithelium point to an underlying defect in metaphase progression
Source: Open Biol. 2013 Oct;3(10):130065. doi: 10.1098/rsob.130065 (PMC3814721; doi:10.1098/rsob.130065)
Supplement: Box 1 [file rsob130065supp1.doc]

**Box 1**

**Background on STIL, ASPM, WRD62, and ODF2**

Mutations in human *STIL* were first identified as a cause of MCPH in 2009 . *STIL* is located at chromosomal position 1p32.3, has a 5,019bp coding sequence and contains 18 exons. It encodes a 150KDa cytosolic protein that locates to the centrosome during mitosis. Orthologues have been identified and studied in Drosophila (*Ana-2*) and C.elegans (*SAS-5*) as well as vertebrates including zebrafish and mouse . STIL has been shown to be involved in centriole duplication and to interact with other pericentriolar MCPH proteins, CPAP and CEP152 . In 1999, a *stil*^-/-^ knockout mouse was described, with defects in left-right asymmetry, a reduced number of cells within the developing neural plate and increased apoptosis . More recently, a *stil* loss of function zebrafish mutant, *cassiopeia,* was identified . Two separate homozygous recessive alleles, *csp*^cz65-/-^ and *csp*^cz299-/-^, were shown to cause the same abnormal phenotype involving ventral or dorsal tail curvature, cardiac oedema, mitotic arrest, high levels of apoptotic cell death and embryonic death by 7-10 days post fertilization . In the same study, another *stil* mutant, with a transgenic insertion, *stil* ^hi1262Tg-/-^ displayed a similar but less severe phenotype.

Mutations in *ASPM* are the most common cause of human primary microcephaly . *ASPM* is located at chromosomal position 1q31, has an open reading frame of 10,906bp and contains 30 exons. It encodes a 410 KDa protein that is predicted to contain 4 major domains: a putative microtubule binding domain at the N-terminal end , a calponin homology domain, an IQ-repeat domain containing multiple IQ motifs that are predicted to bind calmodulin and a carboxy-terminal region with no identified domains . *ASPM* is highly conserved between species and it has been hypothesised that the number of IQ domains encoded may correlate with increasing CNS complexity . Mutation of Drosophila *asp* has been shown to cause abnormalities of asymmetric division in the larval brain . More recently, an *Aspm* knockout mouse with a small brain has been described and morpholino-mediated knockdown of zebrafish *aspm* has been shown to cause reduced head size in the developing embryo along with mitotic arrest and increased apoptosis .

*WDR62* mutations were recently identified as the second most common cause of MCPH . *WDR62* is located at chromosomal position 19q13.12, is 4,746 bp in length and consists of 32 exons encoding a 166 kDa protein. The protein contains a conserved region of WD40 repeat domains . These are found in many eukaryotic proteins and are involved in numerous cellular functions including signal transduction and pre-mRNA processing as well as cytoskeletal assembly . Individuals with WDR62 mutations exhibit a wide range of brain malformations in addition to microcephaly .

Although the gene encoding *ODF2* is not directly linked to microcephaly, the protein is linked to several microcephaly/dwarfism proteins and is involved in several cellular processes proposed to be deficient in microcephaly. In the absence of *ODF2*, cell cycle progression is inhibited . Spindle defects are observed, similar to those seen associated with *ASPM* depletion. ODF2 interacts with pericentrin , linking this protein to both CDK5RAP2 and the DNA damage response pathway in which microcephalin is involved . ODF2 is also a centriolar appendage protein like ninein, whose inheritance has been claimed to be critical in asymmetric neurogenic divisions . This suggested that depletion of *odf2* would also lead to a microcephalic phenotype in zebrafish embryos.

REFERENCES:

1. Kumar A, Girimaji SC, Duvvari MR, Blanton SH. Mutations in STIL, encoding a pericentriolar and centrosomal protein, cause primary microcephaly. Am J Hum Genet. 2009;84(2):286-90. Epub 2009/02/14.

2. Tang CJ, Lin SY, Hsu WB, Lin YN, Wu CT, Lin YC, et al. The human microcephaly protein STIL interacts with CPAP and is required for procentriole formation. EMBO J. 2011;30(23):4790-804. Epub 2011/10/25.

3. Stevens NR, Dobbelaere J, Brunk K, Franz A, Raff JW. Drosophila Ana2 is a conserved centriole duplication factor. The Journal of cell biology. 2010;188(3):313-23. Epub 2010/02/04.

4. Pfaff KL, Straub CT, Chiang K, Bear DM, Zhou Y, Zon LI. The zebra fish cassiopeia mutant reveals that SIL is required for mitotic spindle organization. Mol Cell Biol. 2007;27(16):5887-97. Epub 2007/06/20.

5. Izraeli S, Lowe LA, Bertness VL, Good DJ, Dorward DW, Kirsch IR, et al. The SIL gene is required for mouse embryonic axial development and left-right specification. Nature. 1999;399(6737):691-4. Epub 1999/06/29.

6. Stevens NR, Roque H, Raff JW. DSas-6 and Ana2 coassemble into tubules to promote centriole duplication and engagement. Developmental cell. 2010;19(6):913-9. Epub 2010/12/15.

7. Cizmecioglu O, Arnold M, Bahtz R, Settele F, Ehret L, Haselmann-Weiss U, et al. Cep152 acts as a scaffold for recruitment of Plk4 and CPAP to the centrosome. The Journal of cell biology. 2010;191(4):731-9. Epub 2010/11/10.

8. Vulprecht J, David A, Tibelius A, Castiel A, Konotop G, Liu F, et al. STIL is required for centriole duplication in human cells. J Cell Sci. 2012;125(Pt 5):1353-62. Epub 2012/02/22.

9. Bond J, Roberts E, Springell K, Lizarraga SB, Scott S, Higgins J, et al. A centrosomal mechanism involving CDK5RAP2 and CENPJ controls brain size. Nature genetics. 2005;37(4):353-5. Epub 2005/03/29.

10. Saunders RD, Avides MC, Howard T, Gonzalez C, Glover DM. The Drosophila gene abnormal spindle encodes a novel microtubule-associated protein that associates with the polar regions of the mitotic spindle. The Journal of cell biology. 1997;137(4):881-90. Epub 1997/05/19.

11. Bond J, Roberts E, Mochida GH, Hampshire DJ, Scott S, Askham JM, et al. ASPM is a major determinant of cerebral cortical size. Nature genetics. 2002;32(2):316-20. Epub 2002/10/02.

12. Ponting C, Jackson AP. Evolution of primary microcephaly genes and the enlargement of primate brains. Curr Opin Genet Dev. 2005;15(3):241-8. Epub 2005/05/27.

13. Gonzalez C, Saunders RD, Casal J, Molina I, Carmena M, Ripoll P, et al. Mutations at the asp locus of Drosophila lead to multiple free centrosomes in syncytial embryos, but restrict centrosome duplication in larval neuroblasts. J Cell Sci. 1990;96 ( Pt 4):605-16. Epub 1990/08/01.

14. Pulvers JN, Bryk J, Fish JL, Wilsch-Brauninger M, Arai Y, Schreier D, et al. Mutations in mouse Aspm (abnormal spindle-like microcephaly associated) cause not only microcephaly but also major defects in the germline. Proc Natl Acad Sci U S A. 2010;107(38):16595-600. Epub 2010/09/09.

15. Kim HT, Lee MS, Choi JH, Jung JY, Ahn DG, Yeo SY, et al. The microcephaly gene aspm is involved in brain development in zebrafish. Biochem Biophys Res Commun. 2011;409(4):640-4. Epub 2011/05/31.

16. Nicholas AK, Khurshid M, Desir J, Carvalho OP, Cox JJ, Thornton G, et al. WDR62 is associated with the spindle pole and is mutated in human microcephaly. Nature genetics. 2010;42(11):1010-4. Epub 2010/10/05.

17. Hussain MS, Baig SM, Neumann S, Nurnberg G, Farooq M, Ahmad I, et al. A truncating mutation of CEP135 causes primary microcephaly and disturbed centrosomal function. Am J Hum Genet. 2012;90(5):871-8. Epub 2012/04/24.

18. Bhat V, Girimaji SC, Mohan G, Arvinda HR, Singhmar P, Duvvari MR, et al. Mutations in WDR62, encoding a centrosomal and nuclear protein, in Indian primary microcephaly families with cortical malformations. Clin Genet. 2011;80(6):532-40. Epub 2011/04/19.

19. Bilguvar K, Ozturk AK, Louvi A, Kwan KY, Choi M, Tatli B, et al. Whole-exome sequencing identifies recessive WDR62 mutations in severe brain malformations. Nature. 2010;467(7312):207-10. Epub 2010/08/24.

20. Yu TW, Mochida GH, Tischfield DJ, Sgaier SK, Flores-Sarnat L, Sergi CM, et al. Mutations in WDR62, encoding a centrosome-associated protein, cause microcephaly with simplified gyri and abnormal cortical architecture. Nature genetics. 2010;42(11):1015-20. Epub 2010/10/05.

21. Soung NK, Park JE, Yu LR, Lee KH, Lee JM, Bang JK, et al. Plk1-dependent and -independent roles of an ODF2 splice variant, hCenexin1, at the centrosome of somatic cells. Developmental cell. 2009;16(4):539-50. Epub 2009/04/24.

22. Buchman JJ, Tseng HC, Zhou Y, Frank CL, Xie Z, Tsai LH. Cdk5rap2 interacts with pericentrin to maintain the neural progenitor pool in the developing neocortex. Neuron. 2010;66(3):386-402. Epub 2010/05/18.

23. Griffith E, Walker S, Martin CA, Vagnarelli P, Stiff T, Vernay B, et al. Mutations in pericentrin cause Seckel syndrome with defective ATR-dependent DNA damage signaling. Nature genetics. 2008;40(2):232-6. Epub 2007/12/25.

24. O'Driscoll M, Jackson AP, Jeggo PA. Microcephalin: a causal link between impaired damage response signalling and microcephaly. Cell Cycle. 2006;5(20):2339-44. Epub 2006/11/15.

25. Xu X, Lee J, Stern DF. Microcephalin is a DNA damage response protein involved in regulation of CHK1 and BRCA1. The Journal of biological chemistry. 2004;279(33):34091-4. Epub 2004/06/29.

26. Wang X, Tsai JW, Imai JH, Lian WN, Vallee RB, Shi SH. Asymmetric centrosome inheritance maintains neural progenitors in the neocortex. Nature. 2009;461(7266):947-55. Epub 2009/10/16.
