# Supplementary figures and images for: Microcephaly models in the developing zebrafish retinal neuroepithelium point to an underlying defect in metaphase progression
Source: Open Biol. 2013 Oct;3(10):130065. doi: 10.1098/rsob.130065 (PMC3814721; doi:10.1098/rsob.130065)

Supplementary Figure 1

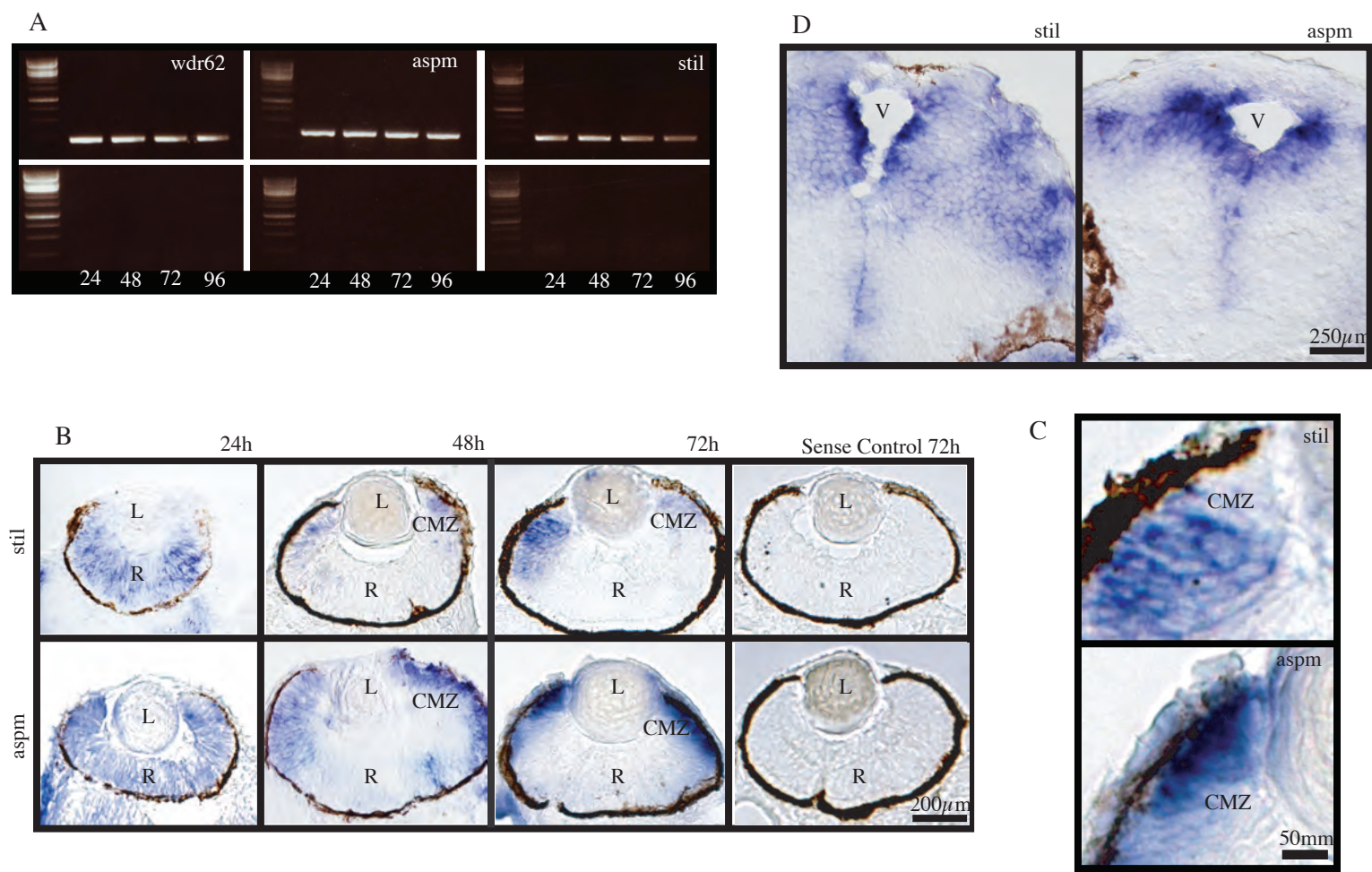

Supplement: Supplementary Figure S1 [file rsob130065supp2.pdf]
