# Supplementary material for: Microcephaly models in the developing zebrafish retinal neuroepithelium point to an underlying defect in metaphase progression
Source: Open Biol. 2013 Oct;3(10):130065. doi: 10.1098/rsob.130065 (PMC3814721; doi:10.1098/rsob.130065)

Supplementary Figure 2

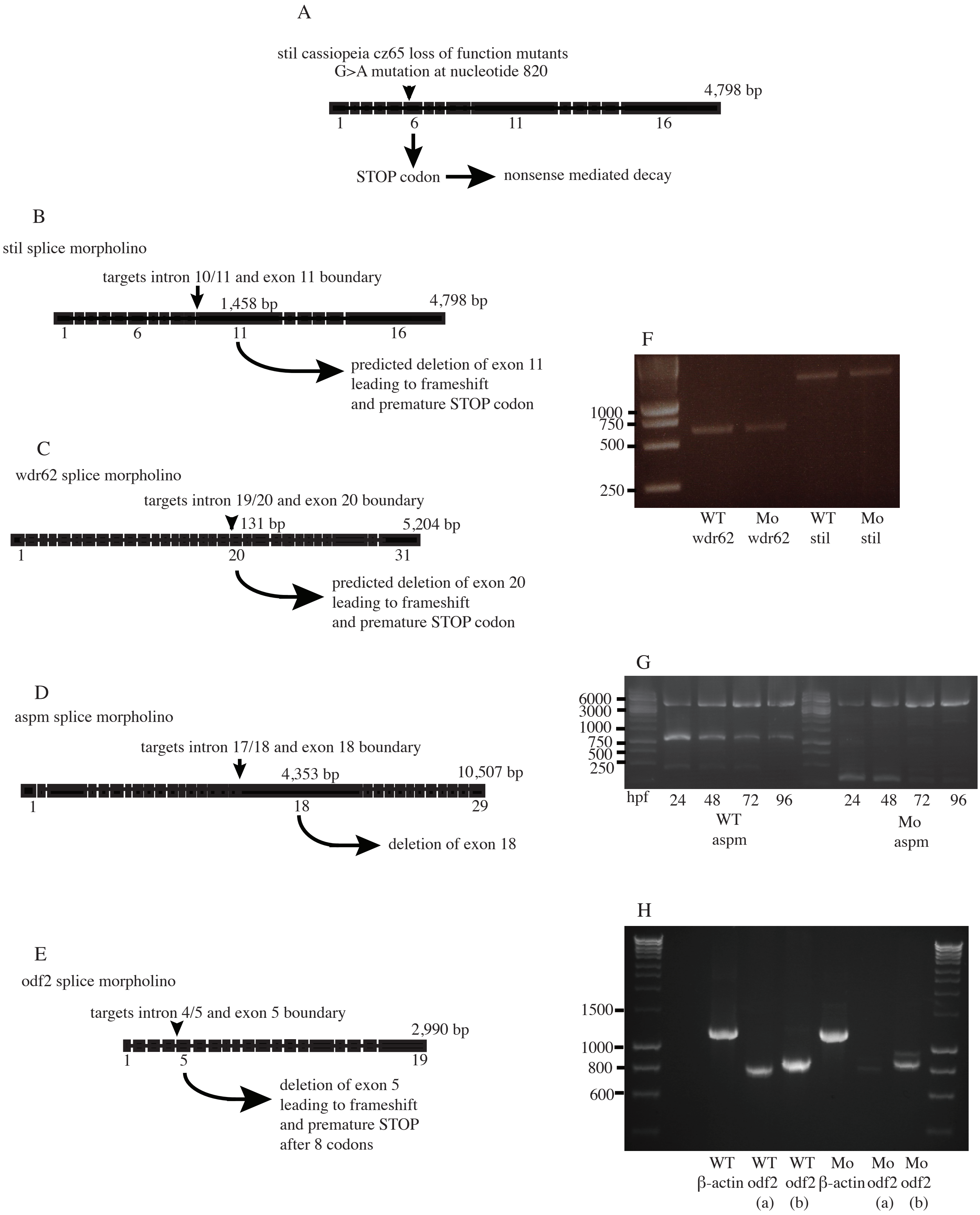

Supplement: Supplementary Figure S2 [file rsob130065supp3.pdf]
