# Supplementary material for: Microcephaly models in the developing zebrafish retinal neuroepithelium point to an underlying defect in metaphase progression
Source: Open Biol. 2013 Oct;3(10):130065. doi: 10.1098/rsob.130065 (PMC3814721; doi:10.1098/rsob.130065)
Supplement: Supplementary figure legends [file rsob130065supp4.docx]

**SUPPLEMENTARY FIGURE LEGENDS**

**Supplementary figure 1.** The MCPH genes *stil*, *aspm* and *wdr62* are expressed in developing zebrafish embryos at early time points including 24hpf, 48hpf, 72hpf and 96hpf. *In-situ* hybridization (ISH) for *stil* and *aspm* demonstrated expression in the retina and brain, with localization to regions of active proliferation. **(A)** RT-PCR demonstrates expression of *wdr62*, *aspm* and *stil* at 24hpf, 48hpf, 72hpf and 96hpf alongside a 1kb DNA ladder. Bands correspond to predicted product sizes - 192bp (*wdr62*), 314bp (*aspm*) and 309bp (*stil*). Control PCR reactions (minus the RT step) are shown for each time point and condition, directly below each corresponding gel. **(B)** ISH of zebrafish retinal sections, using DIG-labelled antisense probes, demonstrates expression patterns of *stil* and *aspm* at 24hpf, 48hpf and 72hpf. Expression of *stil* is seen throughout the neural retina at 24hpf but by 48hpf expression occurs almost exclusively at the ciliary marginal zone (CMZ). Continued CMZ-specific expression of *stil* can be seen at 72hpf. Similarly, *aspm* is expressed throughout the neural retina at 24hpf. However, at 48hpf expression occurs mainly at the CMZ and in adjacent retinal tissue and by 72hpf strong *aspm* expression is seen in the CMZ, with weak to no staining elsewhere in the retina. Sense probe hybridization was performed as a control (shown here for each gene at 72hpf). **(C)** CMZ staining at 72hpf for both *stil* and *aspm* shown in high magnification. **(D)** ISH of zebrafish embryo brain sections at 72hpf demonstrates *stil* and *aspm* expression in the periventricular regions, where progenitor cells continue undergoing mitotic cell division.

**Supplementary figure 2.** The nature of the *stil*^cz65-/-^ mutation is shown here in schematic form along with the binding sites and predicted actions of splice morpholinos used. Gel electrophoresis demonstrates band shifts observed following morpholino injection. **(A)** In *stil*^cz65-/-^ loss of function mutants a G>A mutation at nucleotide 820 results in a premature STOP codon and likely nonsense-mediated decay ([1](#_ENREF_1)). Splice site morpholinos were designed to target **(B)** the junction between intron 10/11 and exon 11 of zebrafish *stil*, **(C)** the junction between intron 19/20 and exon 20 of zebrafish *wdr62,* **(D)** the junction between intron 17/18 and exon 18 of zebrafish *aspm* and **(E)** the junction between intron 4/5 and exon 5 of zebrafish *odf2*. The predicted effects of these splice morpholinos are noted in each corresponding schematic. **(F)** Gel electrophoresis of RT-PCR products from *wdr62* morpholino or *stil* morpholino treated embryos versus untreated controls. In both cases it was predicted that the deletion of exon 11 (*stil*) and exon 20 (*wdr62*) would lead to a frameshift and downstream premature STOP codon. The lack of smaller sized products due to exon skipping may indicate that the resulting shortened mRNA is unstable and degraded. **(G)** Gel electrophoresis demonstrates a change in the pattern of RT-PCR products in the *aspm* morphants. Two bands are seen in control (4,672 bp as predicted, and a second smaller band of ~800bp, presumably from a splice variant). In morphants, this ~800bp band disappears. **(H)** Gel electrophoresis demonstrates band alterations caused by the *odf2* morpholino (at 24hpf). Band **(a)** is the result of a forward primer in exon 1 and reverse primer in exon 6 (size in control, 773bp; in *odf2* morphant, predicted 585bp). In the morphant, no band shift is seen but significant depletion of the band occurs (lane 7 vs. lane 4). Band **(b)** is the result of a forward primer in exon 1 and a reverse primer in exon 8 (size in control 797bp, predicted size in morphant 609bp). In the morphant, an additional larger band is seen (lane 8 vs. lane 5). This was interpreted as cryptic splice sites being revealed in intron 7.

1. Pfaff KL, Straub CT, Chiang K, Bear DM, Zhou Y, Zon LI. The zebra fish cassiopeia mutant reveals that SIL is required for mitotic spindle organization. Mol Cell Biol. 2007;27(16):5887-97. Epub 2007/06/20.
