# Supplementary material for: Microcephaly models in the developing zebrafish retinal neuroepithelium point to an underlying defect in metaphase progression
Source: Open Biol. 2013 Oct;3(10):130065. doi: 10.1098/rsob.130065 (PMC3814721; doi:10.1098/rsob.130065)
Supplement: Supplementary Table 1 [file rsob130065supp5.doc]

**Table 1** *standard dose used (unless stated otherwise)

| **Morpholino** | **Sequence** | **Dose** | **Predicted action** |
| --- | --- | --- | --- |
| Standard Control Mo | 5’-CCTCTTACCTCAGTTACAATTTATA-3’ | 1ng -*8ng | No specific binding to zebrafish DNA |
| anti-*stil* splice Mo | 5’-GAGTCGTCCTGAAAGAATAAAGCAT-3’ | 1ng-8ng  *6ng | Deletion of exon 11 within zebrafish *stil* leading to frameshift and premature STOP codon |
| anti-*aspm* splice Mo | 5’-ACGTTTTCTGGTGGAACACAAGAAA-3’ | 1ng – 8ng  *2ng | Deletion of exon 18 within zebrafish *aspm* |
| anti-*wdr62* splice Mo | 5’-GTCTCTCGTCTGTCAAATTGGAAAA-3’ | 8ng | Deletion of exon 20 within zebrafish *wdr62* leading to frameshift and premature STOP codon |
| anti-*p53* Mo | 5’-GCGCCATTGCTTTGCAAGAATTG-3’ | 4ng | Blocks translation of zebrafish *p53* |
| anti-*odf2* Mo | 5' CTTGTTCTCACTGATTTCTTCATGC 3’ |  | Blocks translation of zebrafish *odf2* |
| anti-*odf2* splice Mo | 5' GAAGCTCTACCTGTAAACACAAACA 3' |  | Deletion of exon 5 of zebrafish *odf2* and premature STOP codon in exon 6 |
