# Supplementary material for: Microcephaly models in the developing zebrafish retinal neuroepithelium point to an underlying defect in metaphase progression
Source: Open Biol. 2013 Oct;3(10):130065. doi: 10.1098/rsob.130065 (PMC3814721; doi:10.1098/rsob.130065)
Supplement: Supplementary Table 2 [file rsob130065supp6.doc]

**Table 2**

| **Primer pair** | **Sequences** | **Predicted product size** | **Post-Mo predicted product size** | **Special RT- PCR conditions** |
| --- | --- | --- | --- | --- |
| *stil* | Fw-5'-CACCCGTACGACTTTCGATT-3'  and  Rv-5'-GACGGTGAGCCAAACGTAAT-3' | 309bp | n/a |  |
| *aspm* | Fw-5'-GCTTTTTAGACGTCGCAAGG-3'  and  Rv-5'-AAATGGTGTGTTGCTCCA-3' | 314bp | n/a |  |
| *wdr62* | Fw-5'-GTGGCAGAGGTCCAGTGTCATAAGT-3'  and  Rv-5'-ACATGTCTGTTGCCTGCAGTTACAA-3' | 221bp | n/a |  |
| *stil* exon 10-Fw and exon 12-Rv | Fw-5’- TGTTTGAGGAAGTGCTCTCACAGTC – 3’  and  Rv-5’- ACAGCAATACTGACACTTGTGCGTTTA -3’ | 1,300bp | 162bp* | Extension time of 2 mins. Annealing temp 62°C |
| *aspm* exon 17-Fw and exon 19-Rv | Fw-5’- CTGGCTGCCACTAAAATACAAGCAC -3’  and  Rv-5-‘ ATTGTCGCAGCTGAAGAAACTTTGA -3’ | 4,672bp | 319bp |  |
| *wdr62* exon 18-Fw and exon 22-Rv | Fw-5’- GCATCTCATCACTGTGTCTGGTGAC -3’  and  Rv-5’- TCTTTTCCTCCTCCTCCTCTTCTCC -3’ | 605bp | 474bp* | Extension time 1 min. Annealing temp 63.5°C |
| *odf2* exon 1-Fw and exon 6-Rv or exon 8-Rv | Fw - 5'-TGCACGTTTTGCTCATATCGGCGTTTGAAT-3’  and  Rv1-5’-GTCTGATCCTGACATCTTTTTCTCA-3’  or  Rv2-5’-ACTTGTACTCCTAGACATTTGGCTGAC | 773 bp  797 bp | 585 bp  609 bp |  |
